# Effects of the COVID-19 pandemic on the management of ST-Segment elevation myocardial infarction in Indonesia: a cohort study

**DOI:** 10.12688/f1000research.121526.1

**Published:** 2022-06-08

**Authors:** Eka Ginanjar, Arif Mansjoer, Lusiani Rusdi, Rizky Ramadantie, Hadiki Habib, Lies Dina Liastuti, Sally Aman Nasution, Idrus Alwi, Abdul Rashid Abdul Rahman

**Affiliations:** 1 Division of Cardiology, Department of Internal Medicine, Faculty of Medicine, Universitas Indonesia, Dr Cipto Mangunkusumo Hospital, Central Jakarta, DKI Jakarta, 10430, Indonesia; 2 Integrated Cardiac Service Unit, Dr Cipto Mangunkusumo Hospital, Central Jakarta, DKI Jakarta, 10430, Indonesia; 3 Emergency Unit, Dr Cipto Mangunkusumo Hospital, Central Jakarta, DKI Jakarta, 10430, Indonesia; 4 Division of Respirology and Critical Care, Department of Internal Medicine, Faculty of Medicine, Universitas Indonesia, Dr Cipto Mangunkusumo Hospital, Central Jakarta, DKI Jakarta, 10430, Indonesia; 5 Department of Cardiology & Vascular Medicine, Faculty of Medicine, Universitas Indonesia, Central Jakarta, DKI Jakarta, 10430, Indonesia; 6 An-Nur Specialist Hospital, Bandar Baru Bangi, Selangor, 43650, Malaysia

**Keywords:** COVID-19, STEMI, hospital admission, symptoms-to-hospital time, reperfusion strategy, reperfusion time, mortality rate, MACE

## Abstract

**Background:** ST-segment elevation myocardial infarction (STEMI) is a form of acute coronary syndrome with high mortality rate. Management of STEMI should be performed as soon as possible to prevent further damage. With the emergence of coronavirus disease 2019 (COVID-19), it may face obstacles. To overcome those problems, some changes in policy focusing on fibrinolytic therapy in STEMI patients have been applied. This study aimed to identify the effects of COVID-19 in management of STEMI patients in Indonesia.

**Methods:** This retrospective study was conducted in Dr. Cipto Mangunkusumo Hospital (CMH), the national referral center in Indonesia. We compared data between 2018 to 2019 and 2020 to 2021 as before and during COVID-19 pandemic period, respectively. We analyzed the effects of COVID-19 on STEMI patients' visits to hospital *i.e*., monthly hospital admission and symptoms-to-hospital, management of STEMI *i.e*., the strategies and time of reperfusion, and clinical outcomes of STEMI patients *i.e*., major adverse coronary event and mortality.

**Results:** There was a significant statistically reduced mean of monthly hospital admissions from 11 to 7 (p = 0.002) and prolonged duration of symptoms-to-hospital during COVID-19 from 8 to 12 hours (p = 0.005). There was also a decrease in primary percutaneous coronary intervention (PPCI) procedures during COVID-19 (65.2% vs. 27.8%, p<0.001), which was accompanied by an increased number of fibrinolytic (1.5% vs. 9.5%, p<0.001) and conservative therapy (28.5% vs. 55.6%, p <0.01). Moreover, there was also a prolonged duration of diagnosis-to-wire-crossing time (160 vs. 186 minutes, p = 0.005), meanwhile, percentage of urgent PCI, door-to-needle time, and clinical outcomes were not statistically significant.

**Conclusions:** During COVID-19 pandemic, the number STEMI patients declined in monthly hospital admission, delays in symptoms-to-hospital time, changes in type of reperfusion strategy, and delays in PPCI procedures in CMH. Meanwhile, fibrinolytic time and clinical outcomes were not affected.

## Introduction

ST-segment elevation myocardial infarction (STEMI) as one of the acute coronary syndromes is a condition of acute transmural myocardium ischemia, which causes injury or necrosis of the myocardium with a high mortality rate. ^1^ The management of STEMI including identification, triage, and reperfusion must be performed as soon as possible to prevent further damage to the myocardium. Reperfusion therapy as the main therapy of STEMI plays an important role to improve patients’ clinical outcomes. ^2^ Reperfusion therapy may include fibrinolytic and primary percutaneous coronary intervention (PPCI). However, PPCI is more preferably recommended since it has been proven to provide better clinical results. ^3^

The emergence of coronavirus disease (COVID-19), which is caused by severe acute respiratory syndrome virus 2 (SARS-CoV-2), has made some impact on healthcare services. The high rate of disease transmission of SARS-CoV-2 has created changes in the algorithm of diagnosis and therapy in hospitals. ^4^^–^^6^ Additional screening COVID-19 tests, wearing personal protective equipment, and performing disinfection of medical equipment and wards must be done to prevent the possibility of transmission. ^7^^,^^8^ These conditions have potential to hamper treatment response in managing patients with emergency conditions including STEMI. ^5^^,^^9^ Additionally, reperfusion therapy using PPCI may need more time considering the high risk of transmission whenever the procedure should be done with the unidentified status of their COVID-19 infection. ^10^

In response to those obstacles, some countries have changed their policy on the management of STEMI patients by prioritizing fibrinolytic therapy. ^4^^–^^6^^,^^11^^,^^12^ This policy has also been applied in Dr. Cipto Mangunkusumo Hospital (CMH). Therefore, this study aimed to provide knowledge about the effects of the COVID-19 pandemic on the management of STEMI patients in Indonesia.

## Methods

### Ethics approval and consent

This research was conducted according to the guidelines of the Declaration of Helsinki and approved by the Ethics Committee of the Faculty of Medicine, Universitas Indonesia with number KET-883/UN2.F1/ETIK/PPM.00.02/2021. The Ethics Committee also waived participants consent due to the inconsiderable risk nature of data collection through retrospective datasets already stored on electronic health records, including demographic data, signs and symptoms, pre-existent comorbidity, the duration from symptoms to hospital, the duration of reperfusion strategies, type of reperfusion, and clinical outcomes. The personal information such as medical records numbers and names of patients were deidentified to protect confidentiality.

### Characteristics of study and research participants

This study was a cohort retrospective study conducted at CMH in Jakarta, which serves as the national referral hospital in Indonesia. Information obtained from medical records was collected from all patients with STEMI diagnosis, who had fulfilled inclusion criteria, including patients admitted to CMH who were diagnosed with STEMI according to the diagnostic criteria from European Society of Cardiology (ESC) 2020 and American Heart Association/American College of Cardiology (AHA/ACC) 2014. ^3^^,^^13^ The data were retrieved from medical records taken between March 15, 2018 and March 15, 2020 for “before COVID-19 pandemic” period and between March 16, 2020 and September 14, 2021 for “COVID-19 pandemic” period. ^25^ The cut-off point between before COVID-19 and COVID-19 pandemic period was the same day when the Indonesian government announced the COVID-19 as a national disaster in Indonesia. Before the announcement, there were no policies related to lockdown and other official regulations. The exclusion criteria of this study included patients who came with major adverse coronary events (MACE) and severe comorbidities such as acute stroke, hepatic cirrhosis, chronic inflammation, sepsis, autoimmune disorders, and malignancy. As one of the complications of STEMI, MACE was excluded because it can influence the therapy and outcomes that are observed. The sampling technique was total sampling and calculated using sample size determination in health studies by S.K. Lawanga and S. Lameshow. ^14^ The confidence interval (CI) was 5% and the power of test was 20%. The minimum sample size was 167 subjects.

### Research variables

In this study, we compared the characteristics of STEMI patients before and during COVID-19 pandemic. The outcomes of the study were STEMI patients’ visits to the hospital, including monthly hospital admission and symptoms-to-hospital time; the management of STEMI, including the strategies reperfusion such as PPCI, fibrinolytic, conservative, and urgent PCI; the timeline of reperfusion therapy, including door-to-diagnosis time, door-to-wire crossing time, door-to-needle time, diagnosis-to-wire crossing time, and diagnosis-to-needle time; and the clinical outcomes including MACE and mortality. Patients who underwent PPCI were analyzed specifically about door-to-diagnosis time, door-to-wire-crossing time, diagnosis-to-wire-crossing time, and ischemic time. Patients who administered fibrinolytic agents were analyzed specifically in regards to the door-to-diagnosis time, door-to-needle time, diagnosis-to-needle time, and ischemic time. We also analyzed clinical outcomes including mortality and MACE.

### Definition of each variable

Monthly hospital admission was defined as the number of STEMI patients who visited the emergency department (ED) after experiencing symptoms of STEMI every month, while the strategy of reperfusion included some optional therapies received by the STEMI patients, which were PPCI, fibrinolytic, conservative, and urgent PCI. The definition of symptoms-to-hospital time was the time taken for patients experiencing STEMI symptoms to be admitted to the ED.

Patients who underwent PPCI or received fibrinolytic therapy were assessed for door-to-diagnosis time. This was defined as the time from when the patients came to the ED until they were diagnosed with STEMI. Specifically, door-to-wire crossing time was defined as the duration from the patients came to the ED until they had wire crossing during PPCI, and diagnosis-to-wire crossing time was defined as the duration from when the patients were diagnosed until they underwent wire crossing during PPCI. Meanwhile, door-to-needle time was defined as the duration from the time patients came to the ED until they received fibrinolytic therapy and diagnosis-to-needle time was defined as the duration from the time patients were diagnosed until the fibrinolytic agent was administered. Ischemic time was defined as the duration from the onset of symptoms until the patients received the therapy, either PPCI or fibrinolytic.

### Statistical analysis

Data were collected into excel datasheets and codes were made for further analysis using STATA 15.1 program (StataCorp. 2017) (RRID: SCR_012763). The chi-square (X2) test, Mann-Whitney test, and Fisher’s exact test were performed to compare the variables. Continuous variables were presented as median and mean. Categorical variables are presented as frequency and percentage.

## Results

### Subject characteristics

There were 712 STEMI patients reported during the sampling period. Among those cases, 393 (55%) of them fulfilled inclusion criteria and the study was conducted retrospectively. The first group consisted of 267 STEMI patients who were included in the “before COVID-19 pandemic” period (mean age of 55 years; 84% were male) and the second group consisted of 126 STEMI patients included in the “COVID-19 pandemic” period (mean age was 58 years and 83% were male). ^20^ Risk factors for cardiovascular diseases such as diabetes mellitus, hypertension, dyslipidemia, obesity, acute renal injury, and chronic kidney failure were distributed similarly in both groups. The detailed baseline characteristics are shown in Table 1.

**Table 1.** Basic patient characteristics.

Variables	Before COVID-19 pandemic (n = 267)	COVID-19 pandemic (n = 126)	p value
Age (years), median (IQR)	55 (49-62)	58 (51-63)	0.062
Sex, n (%)			
Male	224 (83.9)	104 (82.5)	0.736
Female	43 (16.1)	22 (17.5)	
Risk factors, n (%)			
Hypertension	162 (60.7)	93 (73.8)	0.011 *
Diabetes	113 (42.3)	60 (47.6)	0.324
Dyslipidemia	101 (37.8)	46 (36.5)	0.801
Acute kidney injury	60 (22.5)	19 (15.1)	0.088
Chronic kidney disease	33 (12.4)	10 (7.9)	0.190
Obesity	19 (7.1)	9 (7.1)	0.992

Abbreviation: n: value, IQR: interquartile range.

* p < 0.05.

### STEMI patients’ visit to the hospital during COVID-19

**Figure 1.**
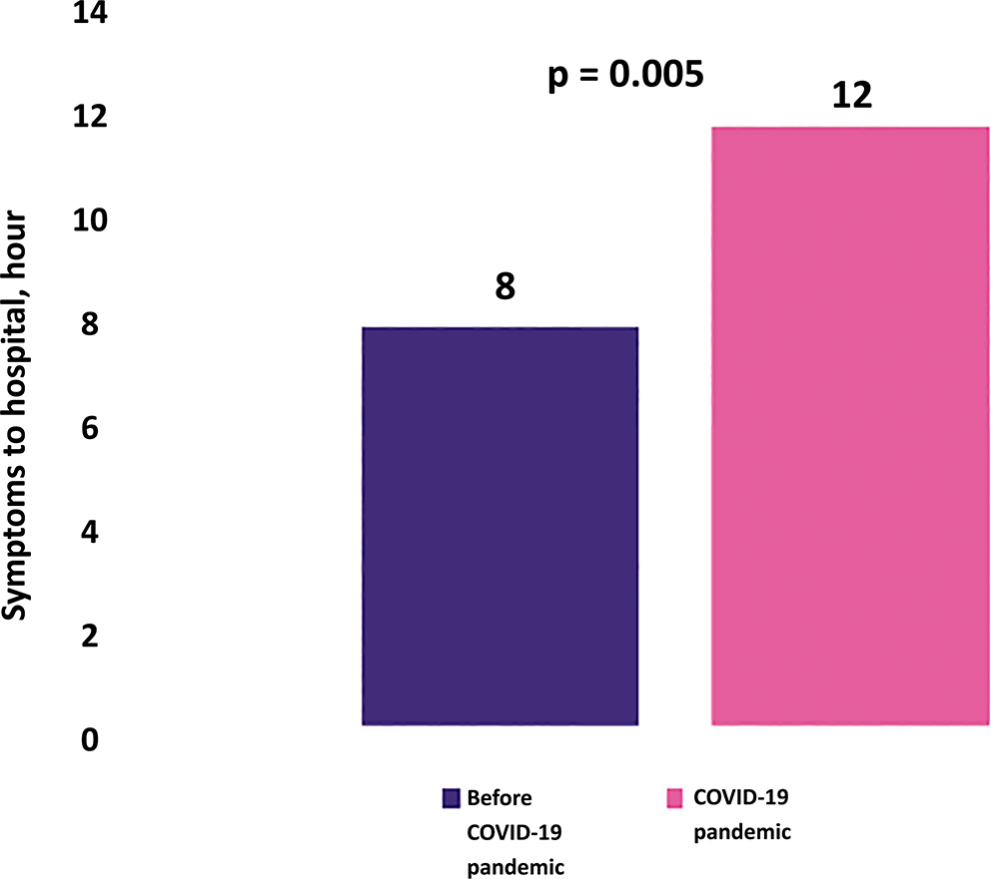
Symptoms-to-hospital before and during COVID-19. The significantly prolonged duration of symptoms-to-hospital was found during COVID-19 pandemic compared to before COVID-19 pandemic.

During the COVID-19 pandemic period, there was a reduced number of monthly hospital admission, *i.e.*, from an average visit of 11 patients to 7 patients (p = 0.002). The percentage of STEMI patients who came with a duration of symptoms-to-hospital of <12 hours had found reduced from 68.1% to 49.2%, while symptoms-to-hospital of >12 hours increased from 31.9% to 50.8% (p < 0.001). The average symptoms to hospital time of STEMI patients before COVID-19 pandemic was 8 hours, while during the COVID-19 pandemic, it prolonged to 12 hours (p = 0.005) ( Figure 1).

### Management of STEMI during COVID-19 pandemic

**Figure 2.**
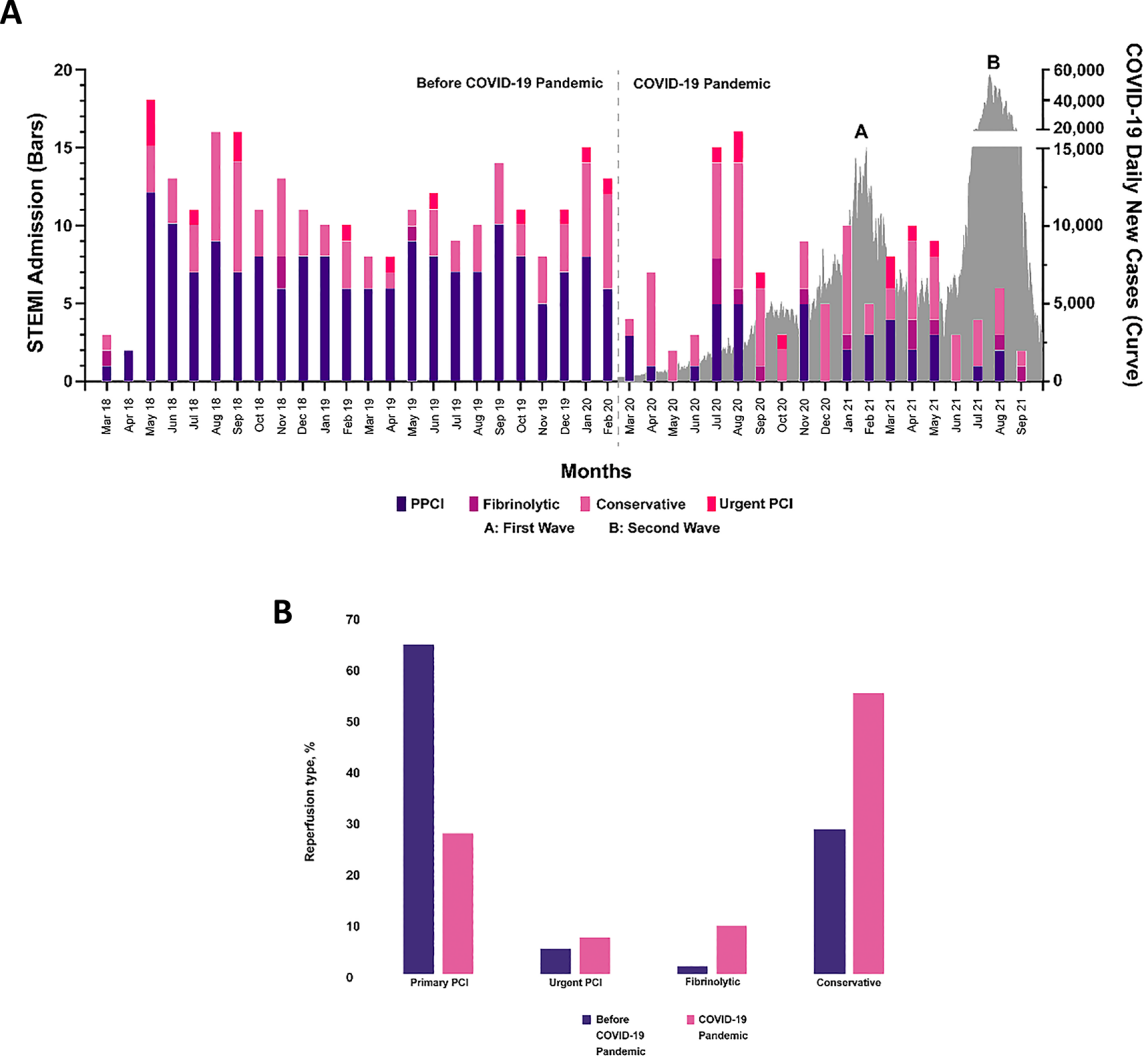
The illustration of COVID-19 pandemic impact on STEMI patients visits and the reperfusion strategy. Panel A shows monthly hospital admission of STEMI patients seems to have decreased during the pandemic and it was accompanied by the strategies of reperfusion. Panel B shows a decreased significantly the percentage of PPCI and fibrinolytic as well as conservative increased, meanwhile, urgent PCI was not statistically affected. Abbreviation: PPCI: percutaneous coronary intervention, urgent PCI: urgent percutaneous coronary intervention. Panel A *i.e.*, the COVID-19 daily new cases has been reproduced with permission from Ministry of Health of the Republic of Indonesia [ https://covid19.go.id/].

We found a statistically significant decrease in the use of PPCI procedure from 65.2% to 27.8% (p < 0.001) and increase in the use of fibrinolytic (1.5% vs. 9.5%, p < 0.001) and conservative therapy (28.5% vs. 55.6%, p < 0.001). However, the number of patients who underwent urgent PCI was not statistically significant (4.9% vs. 7.1%, p = 0.360) ( Figure 2). The door-to-wire crossing time showed a statistically significant increase during COVID-19 pandemic (160 minutes vs. 186 minutes, p = 0.005). Furthermore, we also found the difference in door-to-diagnosis time for PPCI was statistically significant (15 minutes vs. 35 minutes, p = 0.004), but the difference in diagnosis-to-wire crossing time and ischemic time for PPCI were not statistically significant [(136 minutes vs. 134.5 minutes, p = 0.382), (521 minutes vs. 488 minutes, p = 0.421), respectively]. The duration of fibrinolytic therapy, including door-to-needle time (151 minutes vs. 158 minutes, p = 0.953), door-to-diagnosis time for fibrinolytic (16 minutes vs. 12 minutes, p = 0.571), diagnosis-to-needle time (130 minutes vs. 155 minutes, p = 0.851), and ischemic time (321.5 minutes vs. 400.5 minutes, p = 0.599) showed no statistically significant difference.

### Clinical outcomes of STEMI patients during COVID-19 pandemic

The difference in clinical outcomes, including mortality and MACE, were not statistically significant during COVID-19 from 6.7% to 11% (p = 0.220) and from 10.9% to 16.5% (p = 0.107), respectively. The detailed outcomes of this study were shown in Table 2.

**Table 2.** Subject characteristics and distributions.

Study outcomes	Before COVID-19 pandemic (n = 267)	COVID-19 pandemic (n =126)	p value
Monthly hospital admission, mean ± SD	11 ± 4.069	7 ± 4.153	0.002 *
Symptoms-to-hospital, n (%)			
<12 hours	179 (68.1)	61 (49.2)	<0.001 *
≥12 hours	84 (31.9)	63 (50.8)	
Symptoms-to-hospital (hour), median (IQR)	8 (5-13)	12 (5-28.5)	0.005 *
Reperfusion strategies, n (%)			
Primary PCI	174 (65.2)	35 (27.8)	<0.001 *
Urgent PCI	13 (4.9)	9 (7.1)	0.360
Fibrinolytic	4 (1.5)	12 (9.5)	<0.001 *
Conservative	76 (28.5)	70 (55.6)	<0.001 *
Primary PCI (minutes), median (IQR)			
Door-to-diagnosis time	15 (9-32.5)	35 (12.75-65)	0.004 *
Diagnosis-to-wire crossing time	136 (91-165)	134.5 (102.75-206.25)	0.382
Door-to-wire crossing time	160 (121.5-213.5)	186 (149-246)	0.005 *
Ischemic time	521 (390.5-694.5)	488 (329-666)	0.421
Fibrinolytic (minutes), median (IQR)			
Door-to-diagnosis time	16 (9-30.5)	12 (8-19)	0.571
Diagnosis-to-needle time	130 (51.25-204.25)	155 (60-216)	0.851
Door-to-needle time	151 (64.25-225.75)	158 (76.5-218.75)	0.953
Ischemic time	321.5 (139.25-610.25)	400.5 (270.75-646.75)	0.599
Clinical outcomes, n (%)			
Mortality	18 (6.7)	13 (10.3)	0.220
MACE	29 (10.9)	21 (16.7)	0.107

Abbreviations **:** SD: standard deviation, n: value, IQR: interquartile range, PCI: percutaneous coronary intervention, MACE: major adverse coronary event.

* p < 0.05.

## Discussion

This study found that during the COVID-19 pandemic, there was a significant decrease in the average number of monthly of STEMI related hospital admissions compared to before the COVID-19 pandemic period (11 patients vs. 7 patients, p = 0.002). We also found that the STEMI patients who came to the hospital were dominated by those who experienced the onset of symptoms >12 hours earlier with a median time of 12 hours. Some hypotheses can explain those findings: first, during the pandemic, STEMI patients hesitate to visit hospitals as they may be afraid of COVID-19 exposure when they come to the healthcare facilities; second, there is the large-scale social restriction or emergency public activity restriction in Indonesia, therefore, patient access to healthcare facilities becomes limited; third, the lack of cost to visit healthcare facilities and other socio-economic reasons. Although Indonesia has implemented a national health insurance system, some patients have not register to that system yet. Moreover, the cost of some aspects are not covered, such as transportation to CMH because not every patients is transported with ambulance facilities. The accommodation for the families and relatives who accompany the patients is also not covered by this insurance. In COVID-19 pandemic era, the patients could not be accompanied by other relatives due to the risk of transmission thus, the relatives often stayed outside the hospital, in local homestays provided by government and non-government organization.

The reduced number of patients who came to ED during COVID-19 has also occurred in numerous places for extremely varying reasons and these reasons are still speculative. ^9^^,^^15^^–^^18^ A study conducted in France has suggested that the reason for reduced patient visits to ED was that the patients were afraid to get exposed to COVID-19 when they must visit the hospital or they were worried that they would become a burden to healthcare personnel during the difficult time of fighting the pandemic. ^16^ In addition, the factor of persuasion on “stay at home” or lockdown during a pandemic may also have a great contribution to the decrease of patient visits. ^16^ A study in Italy has suggested some other hypotheses on the reduced number of visits, *i.e.* changes to a healthier lifestyle due to the policy to stay at home; therefore, the stress and air pollution levels, which are one of the triggering factors of the coronary event, become less and on the contrary, the feeling of scared being exposed to COVID-19 or misinterpretation of the “stay at home” instruction may also cause patients to not visit hospital although they experience myocardial infarct symptoms. ^17^ Changes in hospital policy that gives a greater priority to COVID-19 patients and executes deferral for patients without any emergency may also become a reason for reduced admission of STEMI patients. ^19^ Lack of education associated with symptoms of heart attack and COVID-19 may also become a factor that can affect the reduced number of visits of STEMI patients who came to the hospital during the COVID-19 pandemic. ^20^

The number of PPCI procedures in CMH showed a significant decrease during pandemic when compared to before the COVID-19 pandemic period (65.2% vs. 27.8%, p < 0.001), while the number of patients who had fibrinolytic (1.5% vs. 9.5%, p < 0.001) and conservative therapy (28.5% vs. 55.6%, p < 0.001) increased. The illustration of COVID-19 daily new cases in Indonesia and STEMI patients with different reperfusion strategies in CMH is depicted in Figure 2. These changes in reperfusion strategies during COVID-19 in STEMI patients were influenced by new policies that were applied in CMH. ^26^ These included a recommendation for using fibrinolytic therapy as first-line treatment for STEMI patients with an onset of <12 hours without any contraindications for fibrinolytic agents and the hemodynamic is stable. The policy is established by considering the status of COVID-19 infection in STEMI patients, which is mostly unidentified before admission and it takes a long time for the COVID-19 test results to be confirmed. Three days were required to get the result of the COVID-19 test and other regions need to wait even longer (up to 8 days). ^21^ Additionally, the absence of a negative-pressure catheterization room and other obstacles may occur because the process of establishing a COVID-19 diagnosis takes place before the reperfusion procedure is performed. Polymerase chain reaction (PCR) tests, the standard diagnostic tool of COVID-19, were scarce, particularly at the beginning of the pandemic in Indonesia when there were only the Ministry of Health’s Research and Development Agency and other three institutions designated as COVID-19 test referral lab. ^21^ Therefore, a significant amount of time was needed to establish the diagnosis of COVID-19, and the PPCI procedure for STEMI patients can be potentially delayed. This policy has also been applied by some countries such as China, Taiwan, Palestine, Iran, and India. ^4^^–^^6^^,^^11^^,^^12^ Fibrinolytic therapy is considered as it can reduce the delay time that occurs in STEMI patients during COVID-19 pandemic.

**Figure 3.**
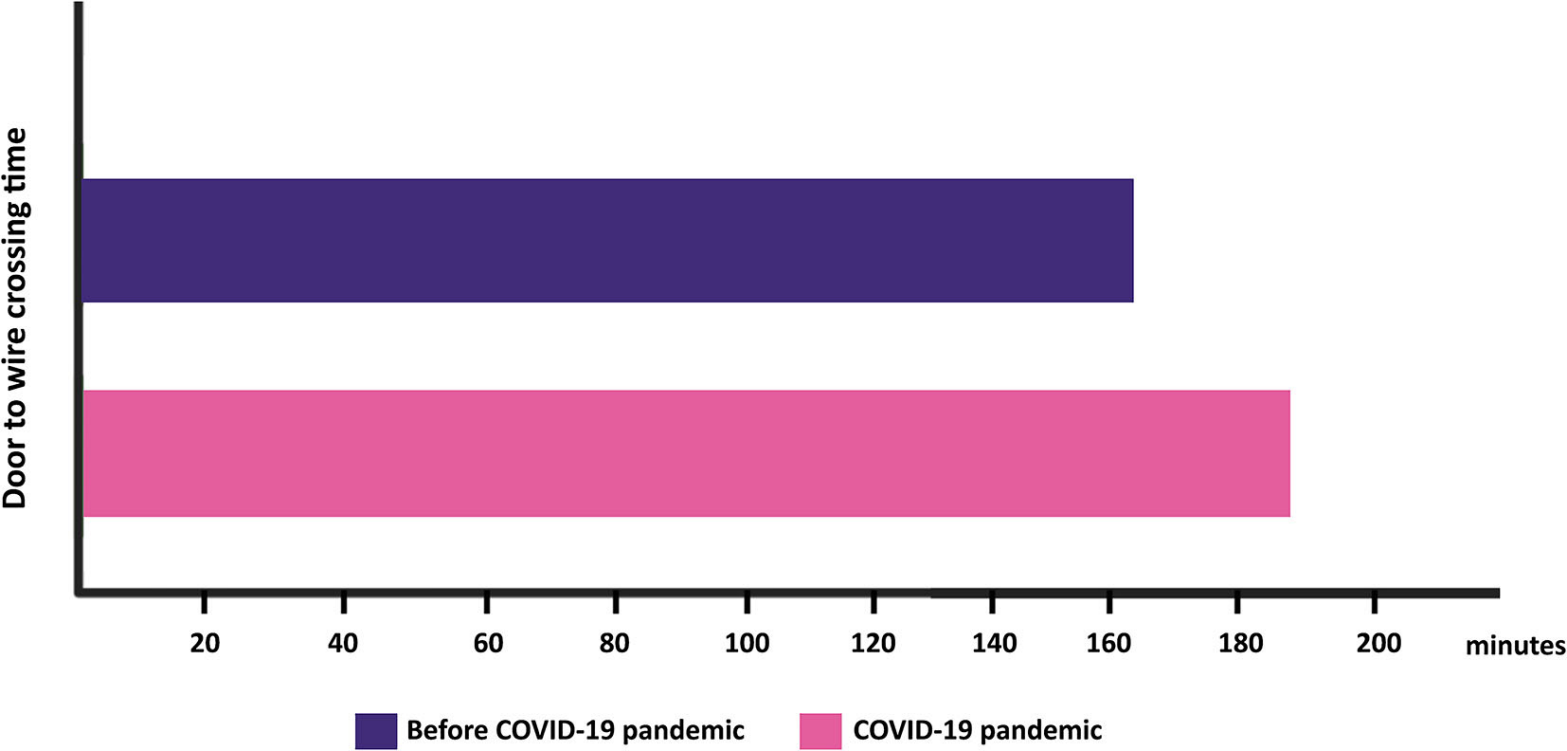
Door-to-wire crossing time before and during COVID-19. The prolonged duration of door-to-wire-crossing time was significantly observed during COVID-19 pandemic compared to before COVID-19 pandemic.

**Figure 4.**
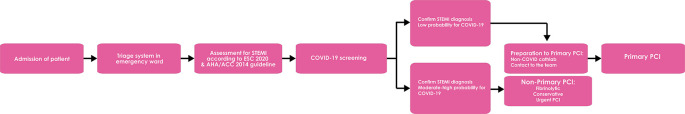
The illustration for diagnosis and therapy of STEMI patients during COVID-19 in Dr. Cipto Mangunkusumo Hospital. According to the policy, there is an additional screening for COVID-19 status for all patients that may delay the treatment. ^26^

In STEMI patients who underwent PPCI, it was observed that the door-to-diagnosis time and door-to-wire crossing time were significantly longer during COVID-19 pandemic ( Figure 3). This can be explained by the occurrence of additional screening COVID-19 tests and procedures to prevent nosocomial infection in ED, from the patients came until the diagnosis can be established. These new procedures include using personal protective equipment, performing disinfection of equipment including electrocardiogram before its usage, and screening the status of COVID-19 infection, *i.e* including radiology examination, laboratory tests, and questionnaire COVID-19 screening ( Figure 4). Meanwhile, the difference in door-to-needle time was found to be not statistically significant. This indicates that basic management for fibrinolytic therapy has not been changed. Patients with STEMI can receive fibrinolytic therapy without waiting for the establishment of COVID-19 status. Right after STEMI was established, patients who fulfill the criteria can receive the fibrinolytic agent. Hence, there are no differences between before and during COVID-19 period for fibrinolytic therapy. The condition of prolonged reperfusion time, both door-to-needle time and door-to-wire crossing time, during COVID-19 pandemic has been reported in previous studies. ^5^^,^^9^^,^^22^^–^^24^ Two studies from China have demonstrated a significantly prolonged time between before and during the pandemic. ^5^^,^^9^ However, other studies in Turkey, England, and Canada have suggested results that are not significantly different, before and during the pandemic. ^22^^–^^24^ These differences may occur since there are some differences in determining policy for reperfusion therapy and the availability of equipment, facility, and catheterization room during the COVID-19 pandemic.

Another finding that we observed was the patient's clinical outcome during hospitalization. In this study, it was found that there was no significant difference in mortality and MACE in STEMI patients during the COVID-19 pandemic period. This indicates that there is no substantial difference in terms of the quality to give treatment for STEMI patients.

Our study was conducted in CMH which serves as the national referral hospital that may provide a general profile of the STEMI patient population in Indonesia during the COVID-19 pandemic.

### Limitations

There is a time gap between before and during the COVID-19 pandemic period regarding the response of the Indonesian government to the COVID-19 pandemic situation that creates uncertainty in determining the cut-point.

## Conclusions

In conclusion, this study found that the number of STEMI patients in CMH declined in monthly hospital admission. There were delays in symptoms-to-hospital time, changes in the type of reperfusion strategy, and delays in door-to-diagnosis time in PPCI and door-to-wire-crossing time. Meanwhile, the duration of fibrinolytic therapy and clinical outcomes were not affected. This study was a retrospective cohort study by analyzing the electronic medical records from our institution. More clinical and basic research is needed in the future to provide the assessment, risk factors, and treatment of STEMI patients during the COVID-19 pandemic.

## Data availability

### Source data

Daily COVID-19 cases in Indonesia: https://covid19.go.id/peta-sebaran (March 2020 to September 2021).

### Underlying data

Figshare: Supplementary File: Effects of COVID-19 Pandemic on the Management of ST-Segment Elevation Myocardial Infarction in Indonesia, https://doi.org/10.6084/m9.figshare.19558969.v2. ^25^

This project contains the following underlying data:

- STEMI Patients Data during COVID-19.xlsx

### Extended data

Figshare: The policy of acute coronary syndrome patients during COVID-19 pandemic in Dr. Cipto Mangunkusumo Hospital, https://doi.org/10.6084/m9.figshare.19728016.v1. ^26^

This project contains the following extended data:

- The policy of acute coronary syndrome management in CMH during COVID-19 pandemic.pdf

Data are available under the terms of the Creative Commons Attribution 4.0 International license (CC-BY 4.0).

## References

[1] Akbar H Foth C Kahloon RA Mountfort S : *Acute ST Elevation Myocardial Infarction.* StatPearls: Treasure Island (FL): StatPearls Publishing;2021 [cited 2021 Aug 23]. Reference Source 30335314

[2] Boersma E Steyerberg EW Van der Vlugt MJ : Reperfusion therapy for acute myocardial infarction. Which strategy for which patient?. *Drugs.* 1998 Jul;56(1):31–48. 10.2165/00003495-199856010-00004 9664197

[3] Thiele H Barbato E Barthelemy O : ESC Guidelines for the management of acute coronary syndromes in patients presenting without persistent ST-segment elevation. 2020;2020:79. 10.1016/j.rec.2021.05.002 34020768

[4] Sadeghipour P Talasaz AH Eslami V : Management of ST-segment-elevation myocardial infarction during the coronavirus disease 2019 (COVID-19) outbreak: Iranian “247” National Committee’s position paper on primary percutaneous coronary intervention. *Catheter. Cardiovasc. Interv.* 2020 Apr 22;97:E346–E351. 10.1002/ccd.28889 32320138 PMC7264551

[5] Xiang D Xiang X Zhang W : Management and Outcomes of Patients With STEMI During the COVID-19 Pandemic in China. *J. Am. Coll. Cardiol.* 2020 Sep;76(11):1318–1324. 10.1016/j.jacc.2020.06.039 32828614 PMC7438071

[6] Daralammouri Y Azamtta M Hamayel H : Recommendations for safe and effective practice of interventional cardiology during COVID-19 pandemic: expert opinion from Jordan and Palestine. *Palest Med. Pharm. J.* (5):65–73.

[7] CDC: Healthcare Workers. Centers for Disease Control and Prevention. 2020 [cited 2021 Aug 8]. Reference Source

[8] Kementerian Kesehatan RI : Panduan Teknis Pelayanan Rumah Sakit pada Masa Adaptasi Kebiasaan Baru. 2020.

[9] Leng WX Yang JG Li XD : Impact of the shift to a fibrinolysis-first strategy on care and outcomes of patients with ST-segment–elevation myocardial infarction during the COVID-19 pandemic—The experience from the largest cardiovascular-specific centre in China. *Int. J. Cardiol.* 2021 Apr;329:260–265. 10.1016/j.ijcard.2020.11.074 33307137 PMC7723441

[10] Han Y Zeng H Jiang H : CSC Expert Consensus on Principles of Clinical Management of Patients With Severe Emergent Cardiovascular Diseases During the COVID-19 Epidemic. *Circulation.* 2020 May 19;141(20):e810–e816. 10.1161/CIRCULATIONAHA.120.047011 32216640

[11] Chopra H Wander GS Kumar AS : Consensus on STEMI Management in the Era of COVID-19. 8. 32738846

[12] Li YH Wang MT Huang WC : Management of acute coronary syndrome in patients with suspected or confirmed coronavirus disease 2019: Consensus from Taiwan Society of Cardiology. *J. Formos. Med. Assoc.* 2021 Jan;120(1):78–82. 10.1016/j.jfma.2020.07.017 32682701 PMC7357505

[13] Amsterdam EA Wenger NK Brindis RG : 2014 AHA/ACC Guideline for the Management of Patients With Non–ST-Elevation Acute Coronary Syndromes. *Circulation.* 2014;130:e344–e426. 10.1161/CIR.0000000000000134 25249585

[14] Lwanga SK Lemeshow S Organization WH : *Sample size determination in health studies: a practical manual.* World Health Organization;1991 [cited 2022 May 5]. Reference Source

[15] Showkathali R Yalamanchi R Sankeerthana MP : Acute Coronary Syndrome admissions and outcome during COVID-19 Pandemic–Report from large tertiary centre in India. *Indian Heart J.* 2020 Nov;72(6):599–602. 10.1016/j.ihj.2020.09.005 33357652 PMC7500406

[16] Mesnier J Cottin Y Coste P : Hospital admissions for acute myocardial infarction before and after lockdown according to regional prevalence of COVID-19 and patient profile in France: a registry study. *Lancet Public Health.* 2020 Oct;5(10):e536–e542. 10.1016/S2468-2667(20)30188-2 32950075 PMC7498416

[17] Campo G Fortuna D Berti E : In- and out-of-hospital mortality for myocardial infarction during the first wave of the COVID-19 pandemic in Emilia-Romagna, Italy: A population-based observational study. *The Lancet Regional Health - Europe.* 2021 Apr;3:100055. 10.1016/j.lanepe.2021.100055 34557800 PMC8454529

[18] Rattka M Dreyhaupt J Winsauer C : Effect of the COVID-19 pandemic on mortality of patients with STEMI: a systematic review and meta-analysis. *Heart.* 2021 Mar;107(6):482–487. 10.1136/heartjnl-2020-318360 33334863

[19] De Rosa S Spaccarotella C Basso C : Reduction of hospitalizations for myocardial infarction in Italy in the COVID-19 era. *Eur. Heart J.* 2020 May 15;ehaa409. 32412631 10.1093/eurheartj/ehaa409 PMC7239145

[20] Hammad TA Parikh M Tashtish N : Impact of COVID-19 pandemic on ST-elevation myocardial infarction in a non-COVID-19 epicenter. *Catheter. Cardiovasc. Interv.* 2021;97(2):208–214. 10.1002/ccd.28997 32478961 PMC7300525

[21] Sucahya PK : Barriers to Covid-19 RT-PCR Testing in Indonesia: A Health Policy Perspective. *J INDO HEALTH POLICY ADM.* 2020 May 10 [cited 2022 May 5];5(2). 10.7454/ihpa.v5i2.3888 Reference Source

[22] Abdelaziz HK Abdelrahman A Nabi A : Impact of COVID-19 pandemic on patients with ST-segment elevation myocardial infarction: Insights from a British cardiac center. *Am. Heart J.* 2020 Aug;226:45–48. 32497914 10.1016/j.ahj.2020.04.022 PMC7211651

[23] Erol MK : Treatment Delays and In-Hospital Outcomes In Acute Myocardial Infarction During The Covid-19 Pandemic: A Nationwide Study. *Anatol. J. Cardiol.* 2020 [cited 2021 Dec 31];24:334–342. 10.14744/AnatolJCardiol.2020.98607 Reference Source 33122486 PMC7724394

[24] Clifford CR Le May M Chow A : Delays in ST-Elevation Myocardial Infarction Care During the COVID-19 Lockdown: An Observational Study. *CJC Open.* 2020 Dec 15;3(5):565–573. 10.1016/j.cjco.2020.12.009 33521615 PMC7834324

[25] Ginanjar E : Supplementary File: Effects of COVID-19 Pandemic on the Management of ST-Segment Elevation Myocardial Infarction in Indonesia. figshare. Dataset. 2022. 10.6084/m9.figshare.19558969.v2 PMC10230174 37265506

[26] Ginanjar E : The policy of acute coronary syndrome patients during COVID-19 pandemic in Dr. Cipto Mangunkusumo Hospital. figshare. Figure. 2022. 10.6084/m9.figshare.19728016.v1

