# Supplementary material for: Effects of the COVID-19 pandemic on the management of ST-Segment elevation myocardial infarction in Indonesia: a cohort study
Source: F1000Res. 2022 Jun 8;11:629. [Version 1] doi: 10.12688/f1000research.121526.1 (PMC10230174; doi:10.12688/f1000research.121526.1)
Supplement: Peer review history

# Peer review: Reviewer response for version 1

**DOI:** 10.5256/f1000research.133395.r172531

**Published:** 2023-05-30

**Authors:** Dafsah Arifa Juzar

**Affiliations:** 1 National Cardiovascular Center Harapan Kita, Jakarta, Indonesia; 2 Cardiology & Vascular Medicine, University of Indonesia, Depok, Indonesia

I congratulate for writing the impact of COVID pandemic on the STEMI performance and outcome. 

 Similar outcome for different reperfusion strategy in this study is novel findings. Several RCTs & registry reported that difference reperfusion strategy lead to different outcome: specifically in hospital and 30 days mortality. In this study, the conservative treatment and fibrinolysis treatment are significantly higher during pandemic as compared to pre-pandemic. Despite higher inferior strategy of reperfusion there is no difference in outcome.

 The novel findings is worth to be elaborated.

 Suggestion to differentiate between the quality of treatment and the difference in stratification or others

 Congratulations once again

Is the work clearly and accurately presented and does it cite the current literature?

Yes

If applicable, is the statistical analysis and its interpretation appropriate?

Yes

Are all the source data underlying the results available to ensure full reproducibility?

No source data required

Is the study design appropriate and is the work technically sound?

Yes

Are the conclusions drawn adequately supported by the results?

Partly

Are sufficient details of methods and analysis provided to allow replication by others?

Yes

Reviewer Expertise:

Acute coronary syndrome; Cardiogenic shock; acute and intensive cardiovascular.

I confirm that I have read this submission and believe that I have an appropriate level of expertise to confirm that it is of an acceptable scientific standard.

# Peer review: Reviewer response for version 1

**DOI:** 10.5256/f1000research.133395.r141551

**Published:** 2022-07-04

**Authors:** Doni Firman

**Affiliations:** 1 Department of Cardiology and Vascular Medicine, Faculty of Medicine, Universitas Indonesia, Jakarta, Indonesia

The COVID-19 Pandemic has significantly impacted the  healthcare system all over the world including Indonesia. This single center report interestingly reported what happened in Indonesia - a developing country in dealt with Covid-19 focused in STEMI management. Ginanjar et al  showed reduced mean of monthly hospital admissions, prolonged duration of symptoms-to-hospital, decrease in primary percutaneous coronary intervention (PPCI) procedures, increased number of fibrinolytic, conservative therapy and prolonged duration of diagnosis-to-wire-crossing time.

 There are some points that I would like to address:

1.  In deciding between fibrinolytic and PPCI, I do not see clearly the explanation or the reason for choosing between those two in ED.
2. Considering prolonged ischemic time, is there data regarding cardiogenic shock?
3. Mortality between before and during pandemic is statistically significant; even the ischemic time was prolonged, is there any explanation?
4. It is better to compare this report to other report from Indonesia ^1^

Is the work clearly and accurately presented and does it cite the current literature?

Yes

If applicable, is the statistical analysis and its interpretation appropriate?

Yes

Are all the source data underlying the results available to ensure full reproducibility?

Yes

Is the study design appropriate and is the work technically sound?

Yes

Are the conclusions drawn adequately supported by the results?

Yes

Are sufficient details of methods and analysis provided to allow replication by others?

Yes

Reviewer Expertise:

interventional cardiology

I confirm that I have read this submission and believe that I have an appropriate level of expertise to confirm that it is of an acceptable scientific standard.

## References

[1] : Decrease in the Number of Patients Presenting With ST-Segment Elevation Myocardial Infarction Across Catheterization Centers in Indonesia During the Coronavirus Disease 2019 Pandemic. *Front Cardiovasc Med* .2021;8: 10.3389/fcvm.2021.676592 676592 10.3389/fcvm.2021.676592 34490361PMC8418056

# Response

**Published:** 2022-07-12

**Authors:** Eka Ginanjar

**Affiliations:** University of Indonesia, Indonesia

Dear Dr. Doni Firman,

 Thank you for reviewing our article. Here are some responses to your points:

1. The reasons for choosing therapy for STEMI patients were enclosed in the 3 ^rd^ paragraph in the discussion section. Generally, patients who came to ER with the onset less than 12 hours and no contraindications for fibrinolytic agents, patients will receive fibrinolytic therapy. We enclose the detail of this policy in the references. ^26^
2. We included cardiogenic shock in the MACE outcome, thus the data is available, but we published it as the general term under “MACE variable”. The data is attached through this link. ^25^
3. Based on our analysis, the mortality was not statistically significant as well as the ischemic time. We concluded that there may be no quality difference for STEMI patients to receive therapy, either fibrinolytic or PPCI.
4. We found the article you enclosed interesting to compare with our study. We'll add the report to our study in the second version.

Kindly look forward to hearing from you in due time regarding our second version and to responding to any further questions and comments you may have.

 Best Regards,

 Eka Ginanjar

 References:

 25. Ginanjar E:Supplementary File: Effects of COVID-19 Pandemic on the Management of ST-Segment Elevation Myocardial Infarction in Indonesia. figshare. Dataset.2022. https://doi.org/10.6084/m9.figshare.19558969.v2

 26. Ginanjar E:The policy of acute coronary syndrome patients during COVID-19 pandemic in Dr. Cipto Mangunkusumo Hospital. figshare. Figure.2022. https://doi.org/10.6084/m9.figshare.19728016.v1
